# Carboxylesterase 2 proteins are efficient diglyceride and monoglyceride lipases possibly implicated in metabolic disease

**DOI:** 10.1016/j.jlr.2021.100075

**Published:** 2021-04-17

**Authors:** Gabriel Chalhoub, Stephanie Kolleritsch, Lisa K. Maresch, Ulrike Taschler, Laura Pajed, Anna Tilp, Helgit Eisner, Philipp Rosina, Benedikt Kien, Franz P.W. Radner, Rudolf Schicho, Monika Oberer, Gabriele Schoiswohl, Guenter Haemmerle

**Affiliations:** 1 Institute of Molecular Biosciences, University of Graz, Graz, Austria; 2 Division of Pharmacology, Otto Loewi Research Center, Medical University of Graz, Graz, Austria

**Keywords:** carboxylesterase 2, lipolytic activity, triglycerides, diglycerides, monoglycerides, liver, small intestine, colon, NAFLD, colitis, 2-AG, 2-arachidonoyl-glycerol, CES2, carboxylesterase 2, DGAT, acyl-CoA:diacylglycerol acyltransferase, DGH, diglyceride hydrolase, DSS, dextran sulfate sodium, MGH, monoglyceride hydrolase, NAFLD, nonalcoholic fatty liver disease, NASH, nonalcoholic steatohepatitis, SEC, size-exclusion chromatography, TGH, triglyceride hydrolase

## Abstract

Carboxylesterase 2 (CES2/Ces2) proteins exert established roles in (pro)drug metabolism. Recently, human and murine CES2/Ces2c have been discovered as triglyceride (TG) hydrolases implicated in the development of obesity and fatty liver disease. The murine *Ces2* family consists of seven homologous genes as opposed to a single *CES2* gene in humans. However, the mechanistic role of Ces2 protein family members is not completely understood. In this study, we examined activities of all Ces2 members toward TGs, diglycerides (DGs), and monoglycerides (MGs) as the substrate. Besides CES2/Ces2c, we measured significant TG hydrolytic activities for Ces2a, Ces2b, and Ces2e. Notably, these Ces2 members and CES2 efficiently hydrolyzed DGs and MGs, and their activities even surpassed those measured for TG hydrolysis. The localization of CES2/Ces2c proteins at the ER may implicate a role of these lipases in lipid signaling pathways. We found divergent expression of *Ces2* genes in the liver and intestine of mice on a high-fat diet, which could relate to changes in lipid signaling. Finally, we demonstrate reduced *CES2* expression in the colon of patients with inflammatory bowel disease and a similar decline in *Ces2* expression in the colon of a murine colitis model. Together, these results demonstrate that CES2/Ces2 members are highly efficient DG and MG hydrolases that may play an important role in liver and gut lipid signaling.

Dysregulated lipid metabolism commonly contributes to the development of metabolic disease. In the liver, excess triglyceride (TG) deposition is a hallmark of nonalcoholic fatty liver disease (NAFLD), the most common liver disease worldwide ranging from benign hepatic steatosis to nonalcoholic steatohepatitis (NASH), which can further progress to liver cirrhosis (1, 2). In addition, high intestinal fat absorption is a risk factor for the development of cardiovascular disease and inflammatory diseases such as inflammatory bowel disease (i.e., ulcerative colitis and Crohn’s disease) (3), which is a risk factor for the development of colon cancer (4).

Low expression of carboxylesterase 2 (CES2) has been implicated in the progression of NAFLD, inflammation, and cancer (5, 6, 7). CES2 is a member of the highly conserved α/β-fold serine hydrolase family. While humans encode a single *CES2* gene, gene duplication (splitting) during evolution led to eight *Ces2* homologous genes (Ces2a–Ces2h and one pseudogene) in mice (7). Human CES2 is expressed in various metabolic organs including the liver, intestine, and kidney and is well known to be involved in the detoxification or metabolic activation of various xenobiotics including drugs, environmental toxicants, and carcinogens (8). Recently, CES2/Ces2c have been shown to hydrolyze endogenous lipid substrates such as TGs and diglycerides (DGs), suggesting a physiological role of CES2/Ces2c in lipid and energy metabolism (7, 9). In line, CES2/Ces2c expression is decreased in the liver of obese humans and mice (6, 10). In mice, Ces2c knockdown causes liver steatosis, whereas overexpression of CES2/Ces2c reverses liver steatosis and improves glucose tolerance on high-fat diet (HFD) (10). In addition, we recently showed that intestine-specific Ces2c overexpression counteracts diet-induced obesity and NAFLD in mice possibly via increasing the chylomicron particle size, which likely accelerates chylomicron lipid uptake in muscles (9).

Based on previous studies, it is hypothesized that Ces2c represents the mouse ortholog for human CES2 (6, 7, 10). However, not all aspects of hepatic CES2 overexpression are phenocopied by murine Ces2c as Ces2c overexpression reverses ER stress in mice, whereas ectopic CES2 expression augments ER stress in the liver (6). To further clarify whether Ces2c or other murine Ces2 members represent the ortholog of CES2, we evaluated neutral lipid (i.e., TG, DG, and monoglyceride [MG]) hydrolase activities for Ces2 protein family members including Ces2a, Ces2b, Ces2c, and Ces2e in comparison with the human CES2 protein. We further determined mRNA levels of *Ces2* gene family members in mice during metabolic stress such as HFD feeding and fasting and evaluated the expression of *CES2*/*Ces2* in colon of humans and mice presenting inflammatory gut disorders. Our study demonstrates that besides their ability to hydrolyze TGs, all investigated CES2/Ces2 proteins are highly active diglyceride hydrolase (DGH) and monoglyceride hydrolase (MGH) challenging the notion that there is a single murine ortholog of human CES2.

## Materials and methods

### Patients

Human colon samples from adult patients with confirmed ulcerative colitis or Crohn’s disease and control subjects were collected as previously described (11). Ethical approval was granted by the ethics committee of the Medical University of Graz and confirmed by the ethics committee of the St John of God Hospital Graz (protocol numbers: 24–281 ex 11/12, 23-015 ex 10/11, 17–291 ex 05/06, and 23-002 ex 10/11) in accordance with the 1964 Declaration of Helsinki and its later amendments. Procedures were carried out in accordance with international guidelines. All participants provided written informed consent. All samples and medical data used in this study were irreversibly anonymized.

### Animals

C57Bl6/J were housed under standard conditions (22[±1]°C, 14:10 h light:dark cycle) in a specific pathogen-free environment with *ad libitum* access to chow diet (11 kJ% fat; V1126, Ssniff Spezialdiäten GmbH) or HFD (45 kJ% fat; D12451, Ssniff Spezialdiäten GmbH). Colitis was induced in C57BL/6J wild-type mice by the addition of 2.5% (wt/vol) of dextran sulfate sodium (DSS; MP Biomedicals) to the drinking water (tap water) as previously described (12). Control animals received tap water only. All mice were kept on DSS-water for 7 days. Body weights were monitored daily. DSS-containing drinking water was also monitored daily to ensure consumption of DSS. After 7 days, mice were sacrificed by cervical dislocation, and the colon was excised, extensively washed with ice-cold PBS, and frozen in liquid N_2_. All animal experiments were approved by the Austrian Federal Ministry for Science, Research and Economy (protocol number BMBWF-66.007/0030-V/3b/2018) and the ethics committee of the University of Graz and were conducted in compliance with the Council of Europe Convention (ETS 123). The age, feeding status, and diets are indicated in the figure legends.

### Cloning and expression of Ces2/CES2 proteins

Murine *Ces2a* (NM_133960.5), *Ces2b* (NM_198171.3), *Ces2c* (NM_145603.2), *Ces2e* (NM_172759.3), *Ces2f* (NM_001079865.2), *Ces2g* (NM_197999.2), *Ces2h* (NM_001272045.2), and human *CES2* (NM_003869.6) were cloned into the pFLAG CMV 5.1 expression vector (Merck, Darmstadt, Germany). Murine *LacZ* cDNA was cloned into the pcDNA4/HisMaxC expression vector (Life Technologies, Carlsbad, CA) as previously described (13). For protein purification, a C-terminal His-tag was added to the respective sequences. Primer sequences and restriction sites are summarized in supplemental Table S1.

For transient protein expression, COS-7 cells (SV-40 transformed monkey embryonic kidney cells; ATCC, catalog no. CRL-1651) were transfected with Flag-tagged *Ces2* constructs, His-tagged *CES2*, and His-tagged *LacZ* as control using Metafectene (Biontex Laboratories GmbH, München, Germany) according to the manufacturer’s instructions. Cells were harvested 48 h after transfection (except for Ces2h: cells were harvested 24 h after transfection) and lysed by sonication in ice-cold buffer A (250 mM sucrose, 1 mM EDTA, 1 mM dithiothreitol, 20 μg/ml leupeptin, 2 μg/ml antipain, 1 μg /ml pepstatin; pH 7.0).

### Measurement of intracellular TG content

COS-7 cells were seeded in 12-well plates and transfected with Flag- or His-tagged *Ces2/CES2* constructs. After 24 h, cells were treated with 0.5 ml of DMEM supplemented with 400 μM oleic acid and [^3^H]-labeled oleic acid as a tracer (0.5 μCi per well). After 1 h of oleic acid incubation, cells were washed twice with PBS and intracellular lipids were extracted two times in 1 ml ice-cold hexane/isopropanol (3:2) by shaking for 10 min at room temperature. The supernatants were collected, and cells were lysed in 0.3 M NaOH/0.1% SDS solution for the determination of protein concentrations by Pierce™ BCA protein assay kit (Thermo Fisher Scientific Inc.). The extracted lipids were evaporated under N_2_ stream and then redissolved in chloroform and separated by TLC using hexane/diethyl ether/glacial acetic acid (70:29:1). The TG bands were stained with iodine and then cut out, and radioactivity was measured by liquid scintillation counting.

### Protein purification with affinity chromatography

For protein purification, His-tagged murine *Ces2a*, *Ces2b*, *Ces2c*, and *Ces2e* and human *CES2* constructs were transfected into Expi293F™ cells (Thermo Fischer Scientific, catalog number: A14527) using ExpiFectamine 293 transfection kit (Thermo Fisher Scientific) according to the manufacturer’s instructions. Seventy-two hours after transfection, the medium enriched with recombinant proteins was collected, centrifuged at 1,200 rpm for 3 min at room temperature, and then filtered through a 0.45 μm filter to remove cell debris. His-tagged Ces2/CES2 proteins were purified by affinity chromatography using ÄKTAprime plus chromatography system and HisTrap HP purification columns (Cytiva, formerly GE Healthcare Life Sciences). The medium filtrate was filled up to 35 ml with the binding (wash) buffer (50 mM Tris/HCl, 500 mM NaCl, 5 mM imidazole, 10% glycerol; pH 7.4). The column was equilibrated with the binding buffer by starting the His-tag purification template program till autozero. After loading the sample, a gradient elution was started using an elution buffer containing high amounts of imidazole (50 mM Tris/HCl, 500 mM NaCl, 500 mM imidazole, 10% glycerol; pH 7.4). Fractions were collected and checked for protein content using the method of Bradford (Bio-Rad, CA) and then pooled and dialyzed overnight at 4°C in the dialysis buffer (50 mM Tris/HCl, 500 mM NaCl, 10% glycerol; pH 7.4). The purified proteins were stored at −80°C until further usage. To check the size and purity of the proteins, 2 μg of the purified proteins was loaded on an SDS-PAGE and stained with Coomassie blue.

### CD measurements

To remove any residual protein impurities from the previous purification, analytical size-exclusion chromatography (SEC) was performed. The protein samples were loaded separately onto a Superdex 200 Increase 10/300GL column (Cytiva) with a flow rate of 0.3 ml/min at 8°C. The running SEC buffer contained 20 mM potassium phosphate, pH 7.4, and 150 mM NaCl. The protein concentration was determined using UV absorption and diluted to 0.2 mg/ml in the SEC buffer. Then CD spectra were acquired for each sample and buffer controls on a JASCO J-1500 spectrophotometer at 20°C using a 1 mm path length quartz cell. CD spectra were recorded from 195 to 260 nm with 0.2 nm data pitch, 100 nm/min, and a response time of 1 s. After buffer subtraction, the secondary structure composition was calculated using the DichroWeb server using the K2D method (14).

### Protein expression

Fifteen micrograms of cell lysate protein was denatured in 1x SDS-loading dye at 95°C, separated by SDS-PAGE and transferred onto a PVDF membrane (pore size: 0.45 μm; Carl Roth, Karlsruhe, Germany) according to standard protocols. Membranes were blocked with 10% nonfat dry milk (Carl Roth) in 1xTST buffer (50 mM Tris HCl, 0.1% Tween 20, 150 mM NaCl; pH 7.4) and then incubated with anti-Flag epitope peroxidase-conjugated antibody (1:8,000; A8592, Sigma-Aldrich) or anti-His (1:3,000; 18184, Abcam). GAPDH (1:20,000; 2118S, Cell Signaling, Danvers, MA) was used as a loading control. Immunoblots were visualized using the Clarity Western ECL plus Western Blotting Detection Reagent (Fisher Scientific) and ChemiDoc Touch Imaging System (Bio-Rad).

### Gene expression

Gene expression was investigated in the liver, duodenum, jejunum, ileum, and colon obtained from *ad libitum*–fed, 2 h-refed, or 16 h-fasted mice fed chow diet or HFD and from human colon samples. RNA was extracted using TRIzol® reagent (Thermo Fisher Scientific) and digested with DNaseI (New England Biolabs). One microgram RNA was reverse-transcribed using the LunaScript™ RT SuperMix Kit (New England Biolabs). Quantitative polymerase chain reaction was performed using the Universal SYBR Green Supermix (Bio-Rad) and the StepOnePlus™ system (Thermo Fisher Scientific). Relative mRNA levels were quantified using the ΔΔCT method with *36B4* as the reference gene. Primer sequences are listed in supplemental Table S2.

### TGH, DGH, and MGH activity assays

For MGH and DGH activity assays, the purified proteins were incubated with 1-octanoyl-*rac*-glycerol (MG C8:0; M2265, Sigma-Aldrich), 1-stearoyl-*rac*-glycerol (MG C18:0; M2015, Sigma-Aldrich), 1-oleoyl-*rac*-glycerol (MG C18:1; M7765, Sigma-Aldrich), 2-oleoyl glycerol (MG C18:1; M2787, Sigma-Aldrich), 2-arachidonoylglycerol (MG C20:4; A8973, Sigma-Aldrich), or 1,2-dioleoyl-*sn*-glycerol (DG C18:1; D0138, Sigma-Aldrich) at 37°C. The substrates were prepared by sonication in the assay buffer (50 mM potassium phosphate buffer, 50 mM KCl; pH 7.4) supplemented with different amounts of CHAPS as the detergent (5 mM for MG and 25 mM for DG). After sonication, FA-free BSA (Sigma-Aldrich) was added to get a final concentration of 1%. Substrates were incubated with purified enzymes in the same buffer at 37°C. The assay was stopped at 75°C for 10 min, and FA release was determined using the commercially available NEFA kit (Wako Chemicals, Neuss, Germany). The triglyceride hydrolase (TGH) assay was performed as previously described (15) with minor modifications. Briefly, the substrate containing triolein (TG C18:1; T7140, Sigma-Aldrich) and radiolabeled triolein [9,10-^3^H] (PerkinElmer Vertriebs GmbH, Traiskirchen, Austria) as the tracer was prepared by sonication in the TGH assay buffer (100 mM potassium phosphate, 50 mM KCl supplemented with 25 mM CHAPS; pH 7.4). FA-free BSA was added after sonication to get a final concentration of 1%. Substrates were incubated with either purified enzymes in the same buffer or protein-enriched cell lysates in buffer A at 37°C. The enzymatic activities were determined by detecting the release of radiolabeled FAs by using a liquid scintillation counter. Assay conditions for TGH, DGH, and MGH are summarized in supplemental Table S3.

TLC was used to separate lipid products after the DGH assay. The substrate containing the mixture of 1,3- and 1,2-DG (DG C18:1; D8894, Sigma-Aldrich) was prepared by sonication in 100 mM potassium phosphate buffer supplemented with 25 mM CHAPS (pH 7.4) and 5% FA-free BSA. Substrate suspensions were incubated with the purified proteins at 37°C, and then, lipids were extracted using the Folch method, evaporated, and redissolved in chloroform:methanol (2:1). The lipid extract was loaded on TLC silica plates (TLC Silica gel 60 aluminum sheets, 20x20 cm, Merck #1.05553.0001, predried for 1 h at 60°C) using CAMAG Automatic TLC Sampler 4. The plate was developed with chloroform:acetone:glacial acetic acid (88:12:1) half way, dried for 10 min at room temperature, again developed full way with toluol, and dried for 30 min at room temperature. The plate was dipped into the charring solution (ethanol 25%, H_3_PO_4_ 10%, CuSO_4_ 5%), dried, and incubated for 20 min at 140°C. Assay conditions are summarized in supplemental Table S3.

### Statistical analyses

Results are expressed as the mean ± SEM. Comparisons were made by unpaired two-tailed Student’s *t* test. *P* values of <0.05 were considered statistically significant.

## Results

### Murine *Ces2* genes are differently expressed in the liver, small intestine, and colon

**Fig. 1:**
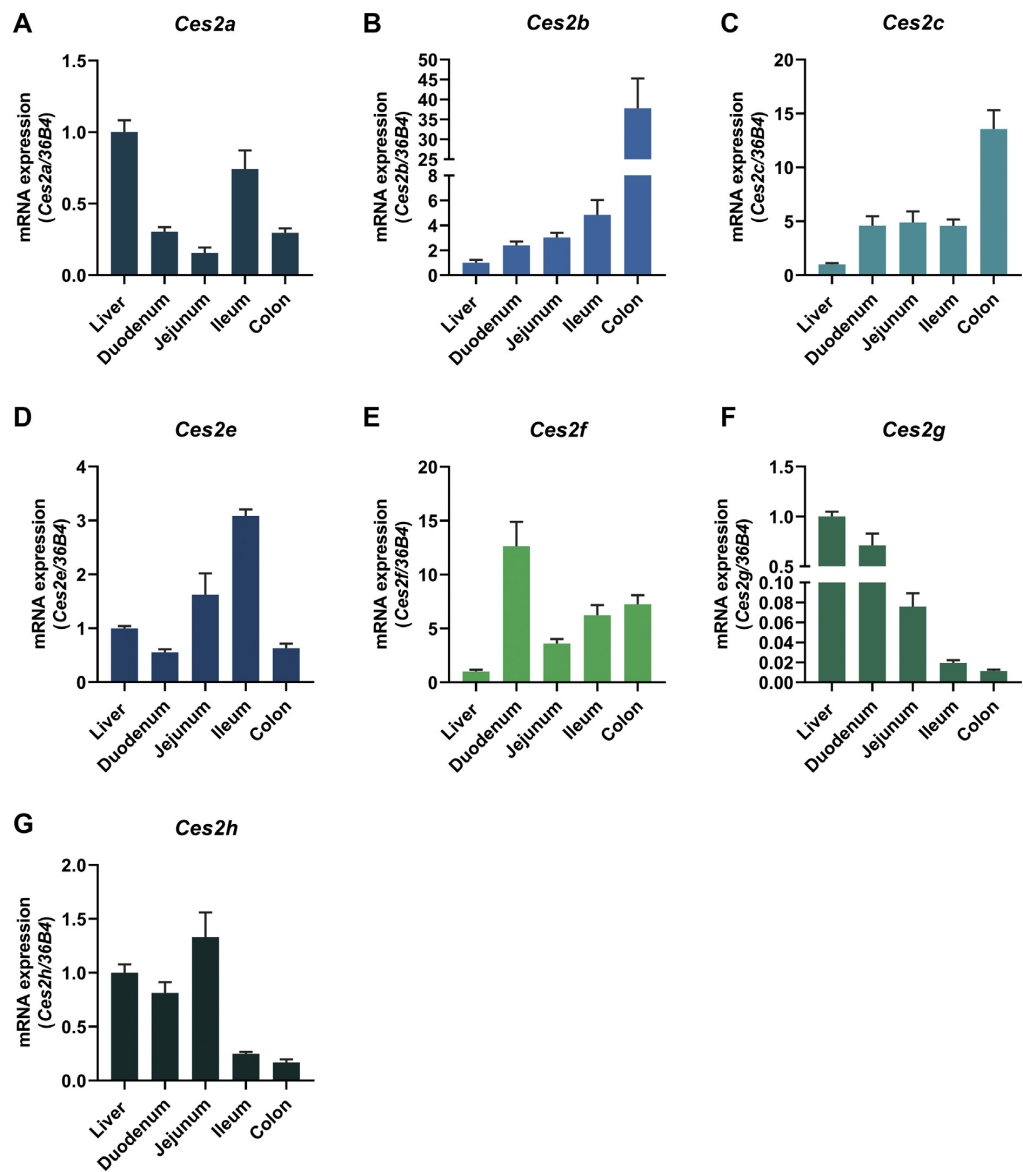
Tissue-specific mRNA expression of murine *Ces2* genes. A–G: Relative mRNA expression determined by quantitative polymerase chain reaction of *Ces2* genes in the liver, duodenum, jejunum, ileum, and colon in mice maintained on a regular chow diet. *36B4* acted as the reference gene, and liver mRNA levels were set to 1 arbitrary unit for each gene (M, 16 weeks of age, 2 h refed, n = 6/group). Data are presented as the means ± SEM. CES2, carboxylesterase 2.

For human CES2, the highest expression is observed in the small intestine followed by the colon and liver (16). To evaluate potential similarities of murine *Ces2* gene expression compared with human *CES2*, we measured mRNA expression of all seven murine *Ces2* genes (*Ces2a*, *Ces2b*, *Ces2c*, *Ces2e*, *Ces2f*, *Ces2g*, and *Ces2h*) in the liver, different sections of the small intestine (the duodenum, jejunum, and ileum), and the colon. *Ces2a* was mainly expressed in the liver and ileum (Fig. 1A), whereas *Ces2b* and *Ces2c* were primarily found in the colon (Fig. 1B, C). In contrast, *Ces2e* was found in the ileum followed by the jejunum and liver (Fig. 1D), whereas *Ces2f* was present in the duodenum and to a lesser extent in the colon, ileum, and jejunum (Fig. 1E). *Ces2g* was mainly expressed in the liver and duodenum (Fig. 1F), and *Ces2h* was similarly expressed in the jejunum, liver, and duodenum (Fig. 1G). Thus, each murine *Ces2* gene exhibits a specific expression profile partly reflecting the expression pattern of human *CES2*.

**Fig. 2:**
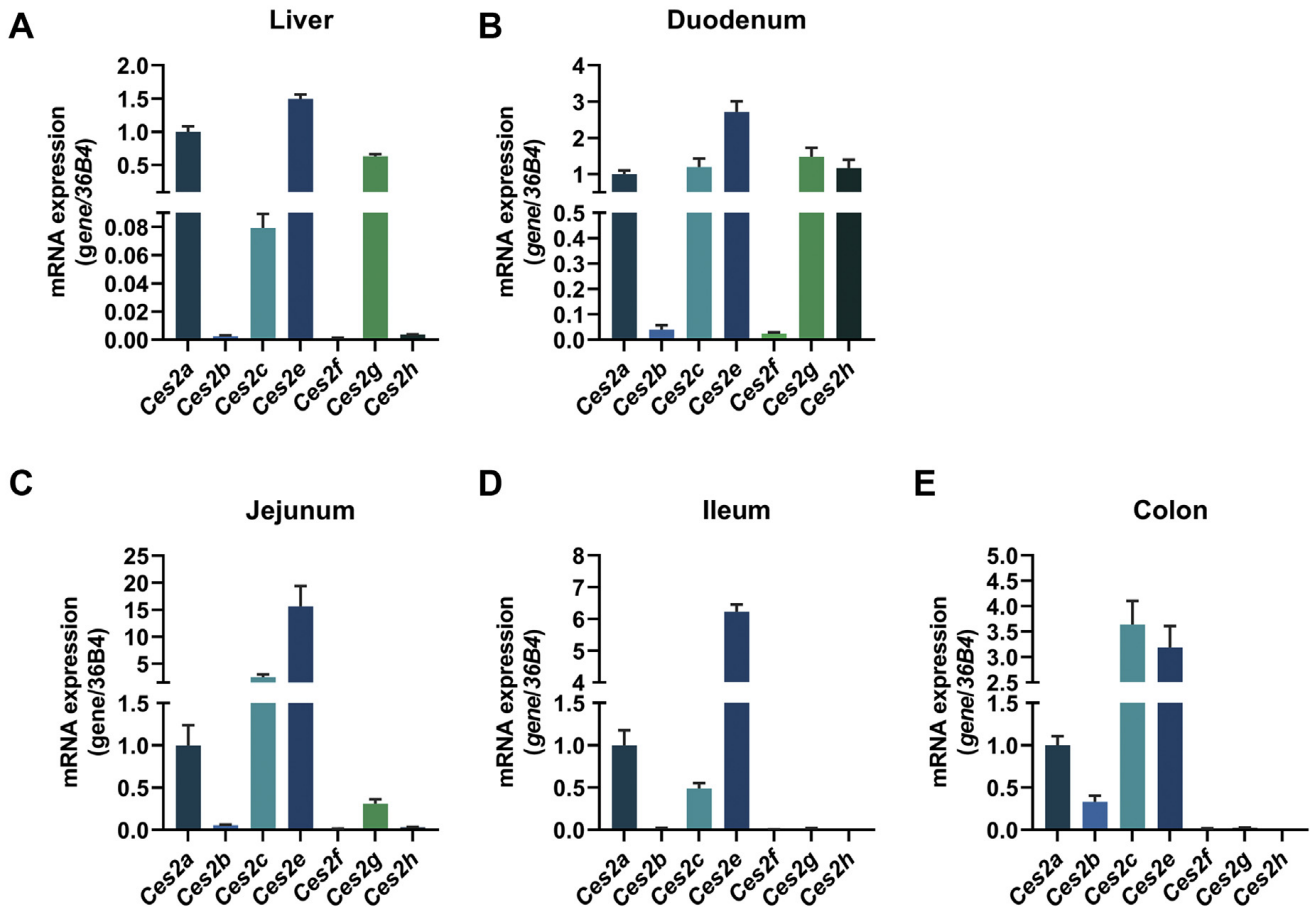
Tissue-specific mRNA expression of murine *Ces2* genes. Relative mRNA expression of *Ces2* genes in the (A) liver, (B) duodenum, (C) jejunum, (D) ileum, and (E) colon in mice kept on chow diet determined by quantitative polymerase chain reaction with *36B4* as the reference gene and *Ces2a* levels set to 1 in each tissue (M, 16 weeks of age, 2 h refed, n = 6/group). Data are presented as the means ± SEM. CES2, carboxylesterase 2.

Because *Ces2* genes were differently expressed among the liver, small intestine, and colon, we also compared their expression within those tissues. In the liver, *Ces2a*, *Ces2e*, and *Ces2g* showed highest expression levels among *Ces2* genes (Fig. 2A). In the duodenum, the highest expression was measured for *Ces2e* followed by *Ces2a*, *Ces2c*, *Ces2g*, and *Ces2h* (Fig. 2B). Likewise, in the jejunum and ileum, *Ces2e* was also the predominantly expressed gene followed by *Ces2c* and *Ces2a* (Fig. 2C, D). In the colon, both, Ces*2c* and *Ces2e*, showed increased expression (Fig. 2E). Notably, in all investigated tissues, the expression of *Ces2e* was the highest or at least among the highest. In contrast, expression levels of *Ces2b*, *Ces2f*, and *Ces2h* were rarely detectable with the exception of *Ces2b* in the colon and *Ces2h* in the duodenum. Thus, the expression pattern of *Ces2* genes varies among and within the liver, small intestine, and colon, suggesting that *Ces2* genes have tissue-specific metabolic functions.

### Specific Ces2 proteins exhibit TGH activity

**Fig. 3:**
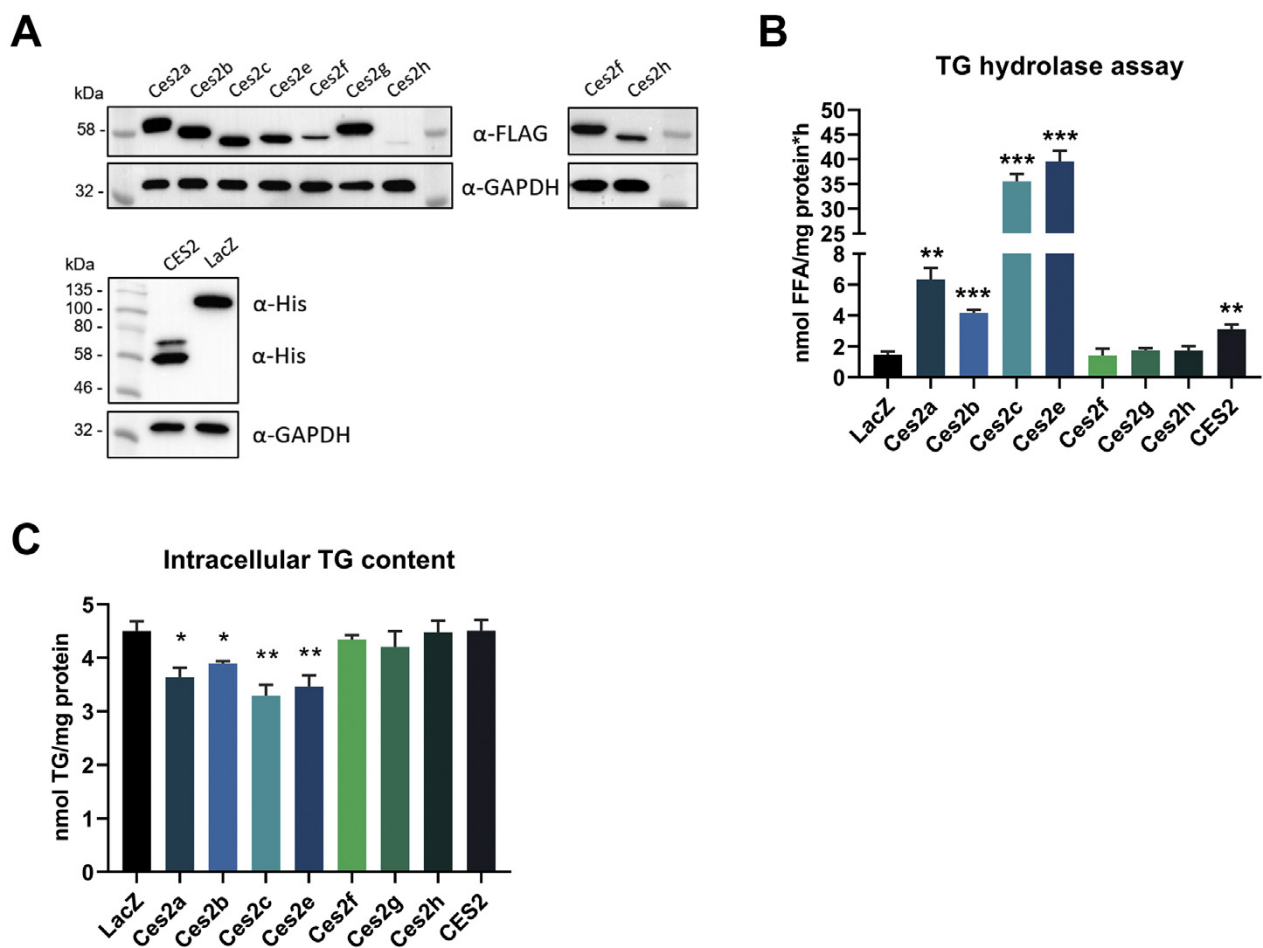
TG hydrolytic activity in COS-7 cell lysates enriched with murine and human Ces2/CES2. A: COS-7 cells were transiently transfected with C-terminally Flag-tagged murine Ces2 cDNA encoding vectors (upper panel; including insert of Ces2f and Ces2h with a longer exposure time), C-terminally His-tagged human CES2, and N-terminally His-tagged LacZ (lower panel). Expression levels were determined by Western blot analysis using anti-Flag or anti-His antibody. GAPDH was used as the loading control. B: Murine and human Ces2/CES2 protein-enriched or LacZ-enriched COS-7 cell lysates were incubated with [^3^H]-labeled triolein to determine TG hydrolytic activity by measuring FA release (n = 3). C, Intracellular TG content of COS-7 cells overexpressing Ces2/CES2. COS-7 cells were loaded with 400 μM oleic acid and [^3^H]-labeled oleic acid as a tracer for 1 h (n = 4). Data are presented as the means ± SEM. Statistical significance was determined by unpaired Student’s *t* test (∗*P* < 0.05; ∗∗*P* < 0.01; ∗∗∗*P* < 0.001 compared with LacZ control). CES2, carboxylesterase 2.

We and others have recently shown that Ces2c and CES2 exhibit TG and DG hydrolytic activities and overexpression of the respective cDNAs interferes with intracellular lipid homeostasis (6, 9, 10). With regard to the sequence homology among *Ces2* gene members, we hypothesized that other *Ces2* gene members may also act as neutral lipid hydrolases. To address this assumption, we overexpressed the cDNA of all murine *Ces2* gene members and human *CES2* in COS-7 cells and measured TGH activities in the respective cell lysates. Western blot analyses confirmed the ectopic expression of Ces2/CES2 proteins in COS-7 cells although the expression of Ces2f and Ces2h was less pronounced (Fig. 3A). Of note, although the predicted molecular weights of the murine Ces2 proteins were comparable (Ces2a: 62.9 kDa, Ces2b: 62.7 kDa, Ces2c: 63.3 kDa, Ces2e: 63 kDa, Ces2f: 63.3 kDa, Ces2g: 63.2 kDa, and Ces2h: 62.9 kDa), the apparent molecular weights of some Ces2 proteins, as estimated from SDS-PAGE, were lower as expected. This might result from posttranslational modifications such as cleavage of the N-terminal ER localization peptide (7). In accordance with the two in-frame transcription start codons in *CES2* (7), overexpression of CES2 resulted in two protein bands detected on the Western blot (Fig. 3A).

As expected, TG hydrolytic activity was increased in cell lysates enriched with Ces2c (24.2-fold) and CES2 (2.1-fold) compared with LacZ expressing control cells. Moreover, cell lysates enriched with Ces2a (4.3-fold), Ces2b (2.8-fold), and Ces2e (27-fold) also showed increased TG hydrolytic activities (Fig. 3B), whereas cell lysates overexpressing Ces2f, Ces2g, and Ces2h exhibited TG hydrolytic activities comparable with LacZ control. Of note, differences in TG hydrolytic activities between the Ces2/CES2 proteins may arise from variable Ces2/CES2 protein levels in the COS-7 cell lysates. Thus, this activity assay clearly emphasized that, despite high sequence similarities among the murine Ces2 proteins, only specific Ces2 proteins exhibit TG hydrolytic activity under the chosen conditions.

Because previous studies showed that overexpression of Ces2c and CES2 reduces endogenous liver TG content (6, 10, 17), we further investigated whether other Ces2 proteins were also capable to alter intracellular TG levels. Therefore, we overexpressed the cDNA of all murine *Ces2* gene members and human *CES2* in COS-7 cells and incubated the cells with oleic acid to measure TG synthesis in the respective cells. Related to increased TG hydrolytic activity, cells overexpressing Ces2a, Ces2b, Ces2c, or Ces2e showed less TG synthesis (i.e., oleic acid incorporation within TGs) compared with the control (Fig. 3C). In contrast and against our expectations, COS-7 cells overexpressing CES2 showed no differences in the intracellular TG content. Together, overexpression of Ces2a, Ces2b, Ces2c, or Ces2e interferes with TG homeostasis in COS-7 cells most likely related to the measured TG hydrolytic activities.

### Murine and human Ces2/CES2 proteins are potent DGH and MGH

To further characterize the Ces2/CES2 proteins that exhibited TG hydrolytic activity in more detail, we purified Ces2a, Ces2b, Ces2c, Ces2e, and CES2 via the mammalian Expi293F system. Successful purification of high amounts of Ces2/CES2 proteins with affinity chromatography (supplemental Fig. S1) was corroborated by SDS-PAGE showing protein bands at the molecular weight comparable with the Western blot analysis of the cell lysates (Fig. 3A).

**Fig. 4:**
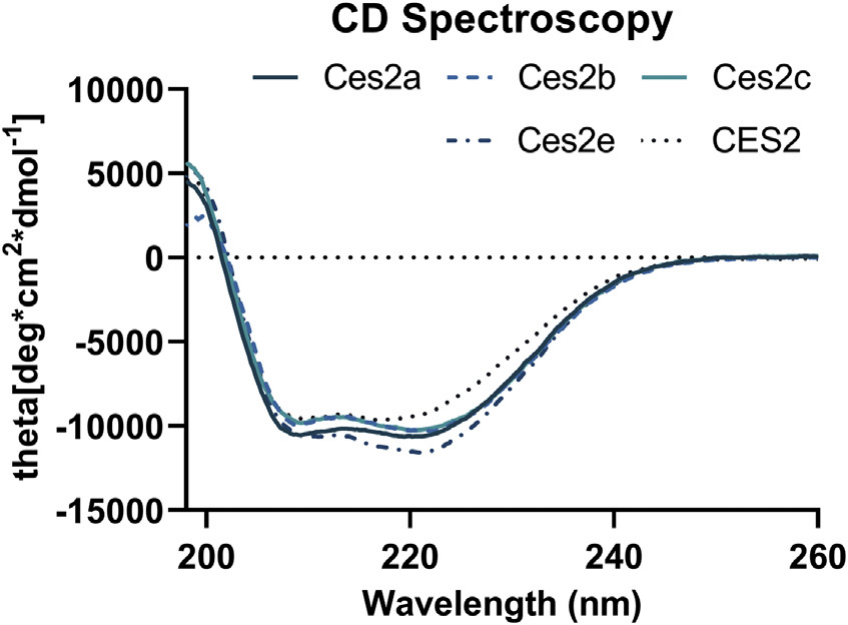
CD spectra of Ces2/CES2 proteins. Secondary structure analyses of Ces2a, Ces2b, Ces2c, Ces2e, and CES2. Overlay of the Ces2/CES2 spectra at room temperature. Each protein was measured at a concentration of 0.2 mg/ml in a buffer containing 20 mM potassium phosphate, pH 7.4, and 150 mM NaCl. CES2, carboxylesterase 2.

In line with high sequence identities (67–88%) of Ces2a, Ces2b, Ces2c, Ces2e, and CES2 (Table 1), CD spectroscopy of the purified Ces2/CES2 proteins revealed comparable CD spectra for Ces2/CES2 proteins with a positive maximum below 200 nm and two minima at 208 nm and 222 nm corresponding to the typical characteristics of a folded protein with the α-helical content (Fig. 4). Analysis of the CD spectra by DichroWeb showed α-helical contents of 31–38% and β-strand contents between 13% and 18% (Table 2).

**Table 1:** Sequence identity of CES2/Ces2 proteins

Protein	Ces2a	Ces2b	Ces2c	Ces2e	CES2
*Ces2a*	100	76.9	76.2	77.4	67.4
*Ces2b*	76.9	100	87.6	77.1	70.9
*Ces2c*	76.2	87.6	100	78.5	71.7
*Ces2e*	77.4	77.1	78.5	100	70.8
*CES2*	67.4	70.9	71.7	70.8	100

CES2, carboxylesterase 2.

Percent sequence identity matrix performed with Clustal Omega (quote Sievers *et al.*, 2011; DOI: 10.1038/msb.2011.75).

**Table 2:** Secondary structure composition of Ces2/CES2

Protein	Helix	Strand	Random Coil
*Ces2a*	33%	19%	48%
*Ces2b*	33%	15%	52%
*Ces2c*	34%	17%	49%
*Ces2e*	38%	13%	49%
*CES2*	31%	18%	51%

CES2, carboxylesterase 2.

The secondary structure content was determined using DichroWeb (quote Whitmore, L. and Wallace, B.A. (2008) Biopolymers 89: 392–400. DOI 10.1002/bip.20853).

**Fig. 5:**
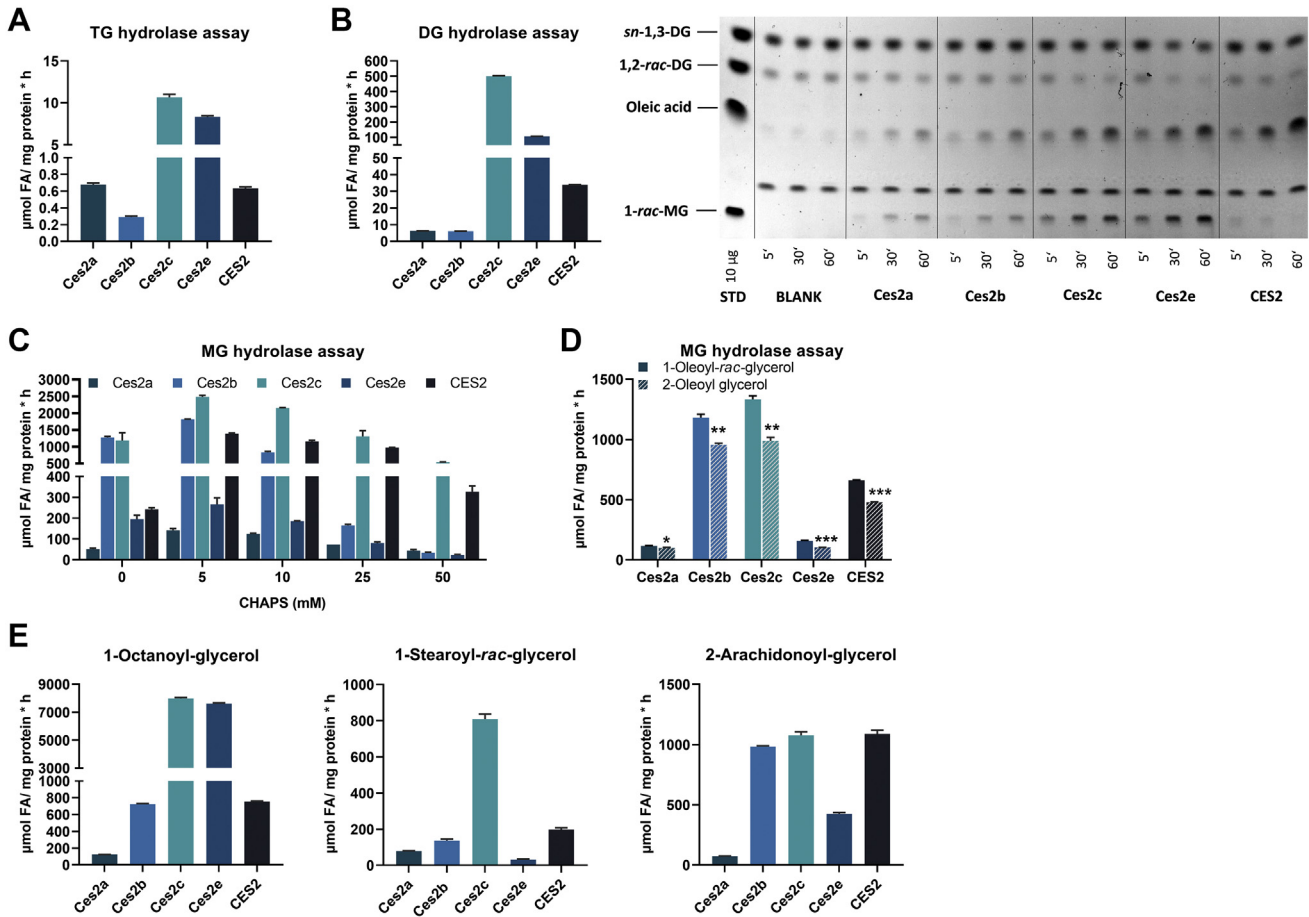
Neutral lipid hydrolase activity assays applying purified recombinant Ces2/CES2. A: Ces2/CES2 were incubated with [^3^H]-labeled triolein to determine TG hydrolytic activity by measuring FA release. B: Left: Ces2/CES2 were incubated with 2-dioleoyl-*sn*-glycerol to determine DG hydrolase activity by measuring the release of FAs. Right: TLC of lipolytic products after DG hydrolysis at indicated time points. C: Ces2/CES2 were incubated with 1-oleoyl-*rac*-glycerol in the presence of different CHAPS concentrations to monitor MG hydrolysis as a measure of released FAs. D: Ces2/CES2 were incubated with 1-oleoyl-*rac*-glycerol or 2-oleoyl glycerol in the presence of 5 mM CHAPS. E: Ces2/CES2 were incubated with 1-octanoyl-*rac*-glycerol (left), 1-stearoyl-*rac*-glycerol (middle), or 2-arachidonoyl-glycerol (right) in the presence of 5 mM CHAPS. Data are presented as the means ± SEM. Statistical significance was determined by unpaired Student’s *t* test (∗*P* < 0.05; ∗∗*P* < 0.01; ∗∗∗*P* < 0.001). CES2, carboxylesterase 2.

Because CES2/Ces2 proteins have high sequence identities along with highly similar secondary structure composition, we were wondering whether purified Ces2/CES proteins exhibit comparable neutral lipid hydrolase activities as well. Similar to increased TG hydrolytic activities in cell lysates (Fig. 3B), purified Ces2c and Ces2e showed increased hydrolytic activity toward TGs compared with Ces2a, Ces2b, and CES2 (Fig. 5A). Likewise, Ces2c and Ces2e exhibited increased DG hydrolytic activity compared with the other Ces2/CES2 proteins (Fig. 5B). Despite differences in their ability to hydrolyze DGs, all Ces2/CES2 proteins exhibited higher activities toward DGs than TGs (Fig. 5A, B). Separation of lipolytic products on a TLC after DGH assays corroborated increased DG hydrolytic activity by reduced levels of 1,3-DG and/or 1,2-*rac*-DG and a time-dependent increase in FAs (oleic acid) especially for Ces2c, Ces2e, and CES2 (Fig. 5B). Notably, incubation with DG substrate increased MG levels of every investigated murine Ces2 protein member but not for CES2 (Fig. 5B), indicating that at least CES2 also exhibits MG hydrolytic activity as well. However, because the substrates for TG and DG hydrolytic activity assays were prepared in the presence of a relatively high concentration of detergent (25 mM CHAPS), we were wondering whether this detergent concentration might impair the MG hydrolytic activity of murine Ces2 proteins. Therefore, we performed MGH activity assays applying different concentrations of CHAPS (Fig. 5C). Indeed, all Ces2/CES2 proteins were capable to hydrolyze MGs in the absence of CHAPS. Ces2b and Ces2c exhibited by far the highest lipolytic activity toward the MG substrate. Interestingly, with increasing CHAPS concentrations, MGH activity became comparable between Ces2b, Ces2c, and CES2 reaching the maximum enzymatic activity at 5 mM CHAPS. Similar results, but with lower levels, were obtained from the remaining Ces2 proteins (Fig. 5C). Further analyses of the regioselectivity of Ces2/CES2 proteins for MGs demonstrated again that Ces2b, Ces2c, and CES2 were the main MGHs among the Ces2/CES2 proteins and that the Ces2/CES proteins preferred to hydrolyze FA ester at the *sn-1* (*sn-3*) position of MG (Fig. 5D). To examine whether the measured MG hydrolytic activity of Ces2/CES2 was dependent on the chain length or saturation grade, we further used 1-octanoyl-glycerol (C8:0), stearoyl-glycerol (C18:0), and 2-arachidonoyl-glycerol (2-AG) (C20:4) as MG substrates (Fig. 5E). Ces2c hydrolytic activity was the highest toward short-chain saturated MG (C8:0) followed by Ces2e, whereas Ces2b and CES2 showed reduced activity. Similar to the hydrolysis of oleoyl-glycerol (C18:1), Ces2c had the highest hydrolytic activity toward stearoyl-glycerol (C18:0), whereas the remaining Ces2/CES2 proteins showed reduced hydrolytic activities toward this saturated MG. In contrast, 2-AG was efficiently hydrolyzed by Ces2b, Ces2c, and CES2. Thus, Ces2/CES2 proteins differ in their substrate selectivity and regioselectivity. However, all Ces2/CES2 proteins hydrolyzed DGs and MGs to a greater extent than TGs, suggesting that Ces2/CES2 proteins could play an important role in lipid signaling processes besides providing FAs as energy substrates and/or lipid intermediates.

### *Ces2* genes are differently expressed in response to metabolic/lipolytic stress

**Fig. 6:**
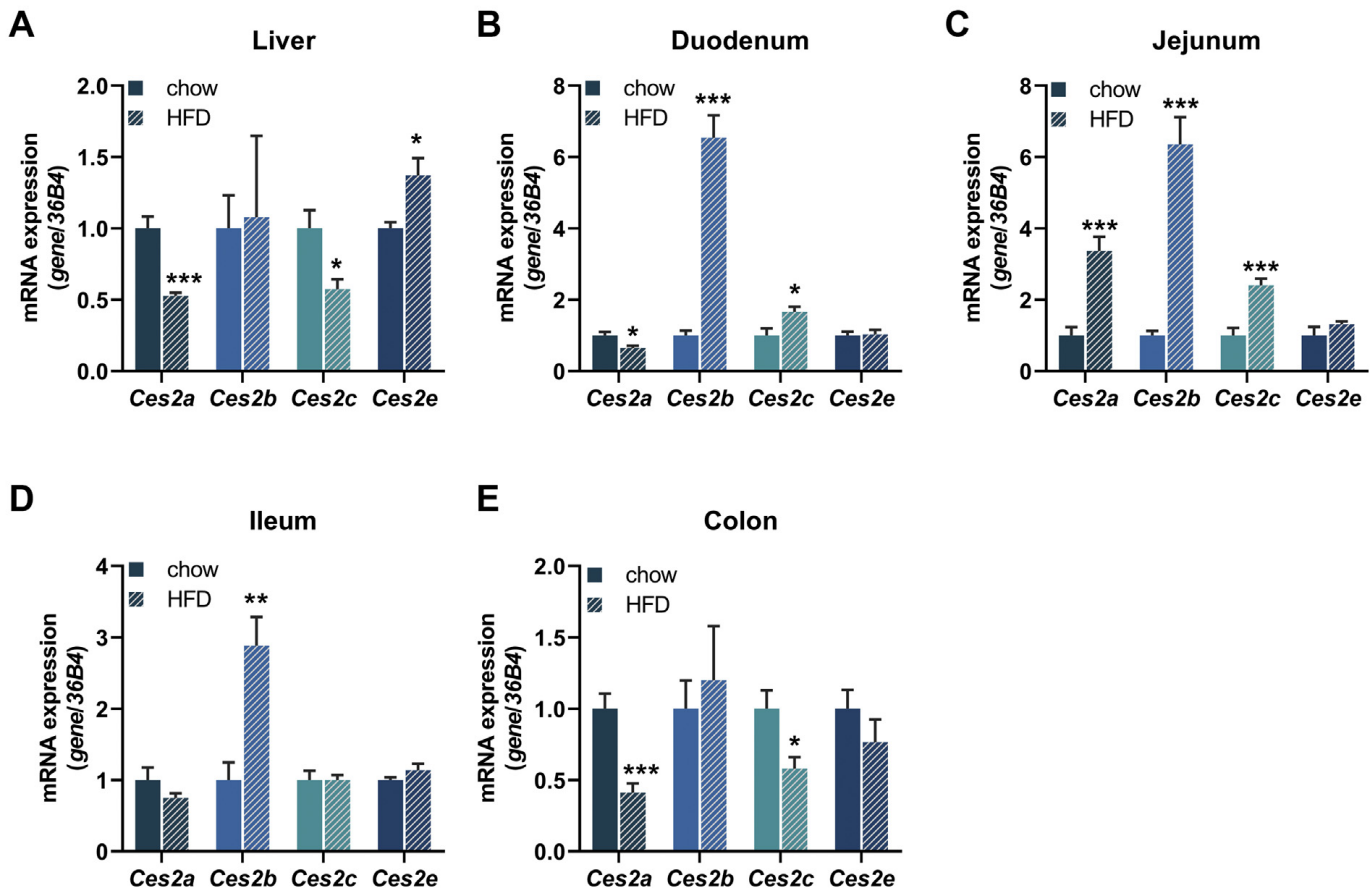
Tissue-specific mRNA expression of murine *Ces2* genes in response to HFD. A–E: Relative mRNA expression of *Ces2a*, Ces2b, *Ces2c*, and *Ces2e* in the (A) liver, (B) duodenum, (C) jejunum, (D) ileum, and (E) colon of chow- or HFD-fed mice determined by quantitative polymerase chain reaction with *36B4* as the reference gene and chow diet levels set to 1 (M, 2 h fed, 16 weeks of age, n = 6/group). Data are presented as the means ± SEM. Statistical significance was determined by unpaired Student’s *t* test (∗*P* < 0.05; ∗∗*P* < 0.01; ∗∗∗*P* < 0.001 for the effect of the diet). CES2, carboxylesterase 2.

Because purified Ces2/CES2 proteins exhibited potent neutral lipid hydrolase activities, we evaluated whether mRNA expression levels were divergently affected by changes in lipid homeostasis caused by metabolic stress such as HFD feeding or fasting. It was already shown that *CES2* expression is reduced in the liver of patients with NASH and obese individuals (6, 10). Therefore, we evaluated the regulation of murine *Ces2* expression in the liver, small intestine, and colon in response to HFD feeding. In the liver, expression of *Ces2a* and *Ces2c* was reduced, whereas *Ces2e* mRNA levels were increased in mice kept on HFD (Fig. 6A). In the small intestine, *Ces2a* expression was downregulated in the duodenum and induced in the jejunum but unchanged in the ileum upon excess fat supply. In contrast, *Ces2b* expression was upregulated in all parts of the small intestine. *Ces2c* expression was upregulated in the duodenum and jejunum but unaffected in the ileum (Fig. 6B–D). In the colon, *Ces2a* and *Ces2c* expression were reduced in HFD-fed mice, whereas *Ces2b* expression was unchanged (Fig. 6E). Interestingly, despite abundant expression of *Ces2e* in the small intestine and colon, HFD feeding did not alter expression levels. Thus, HFD-induced metabolic stress divergently impacts the expression of *Ces2* genes.

**Fig. 7:**
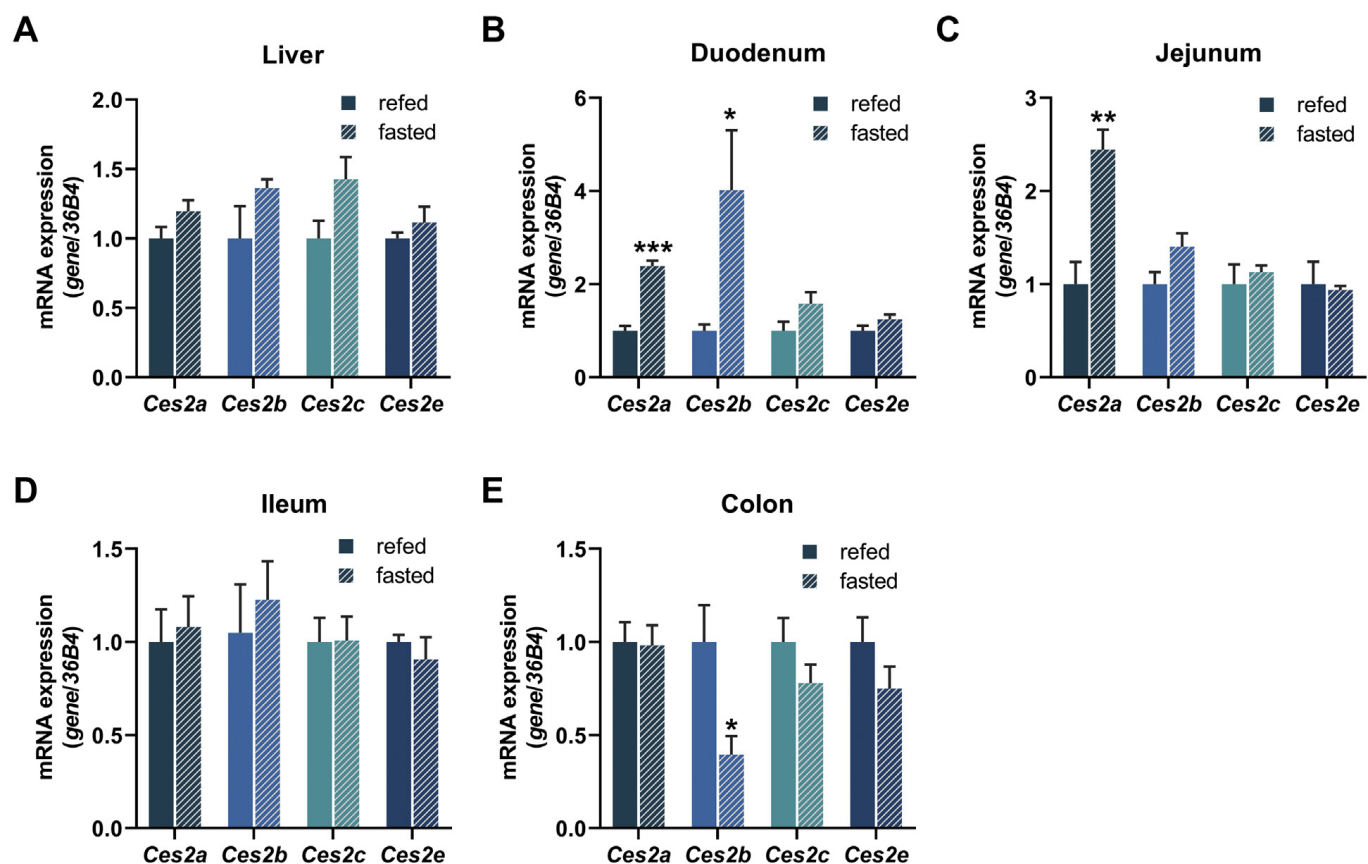
Tissue-specific mRNA expression of murine *Ces2* genes in response to fasting. A–E: Relative mRNA expression of *Ces2a*, *Ces2b*, *Ces2c*, and *Ces2e* in the (A) the liver, (B) duodenum, (C) jejunum, (D) ileum, and (E) colon of chow-fed mice determined by quantitative polymerase chain reaction with *36B4* as the reference gene and chow diet levels set to 1 (M, 2 h fed/16 h fasted, 16 weeks of age, n = 6/group). Data are presented as the means ± SEM. Statistical significance was determined by unpaired Student’s *t* test (∗*P* < 0.05; ∗∗*P* < 0.01; ∗∗∗*P* < 0.001 for the effect of the feeding status). CES2, carboxylesterase 2.

To assess whether acute changes in dietary lipid uptake affected *Ces2* gene expression as well, we determined *Ces2* mRNA expression in the liver, small intestine, and colon in response to fasting (Fig. 7). Notably, fasting had only minor impact on *Ces2* mRNA expression, resulting in an upregulation of *Ces2a* expression in the duodenum and jejunum as well as an increase in *Ces2b* expression in the duodenum but a decrease in the colon. Thus, in contrast to chronic lipid overload, an acute change in lipid availability rarely alters *Ces2* gene expression in the liver, small intestine, and colon.

### *Ces2/CES2* expression is downregulated in the presence of colitis

**Fig. 8:**
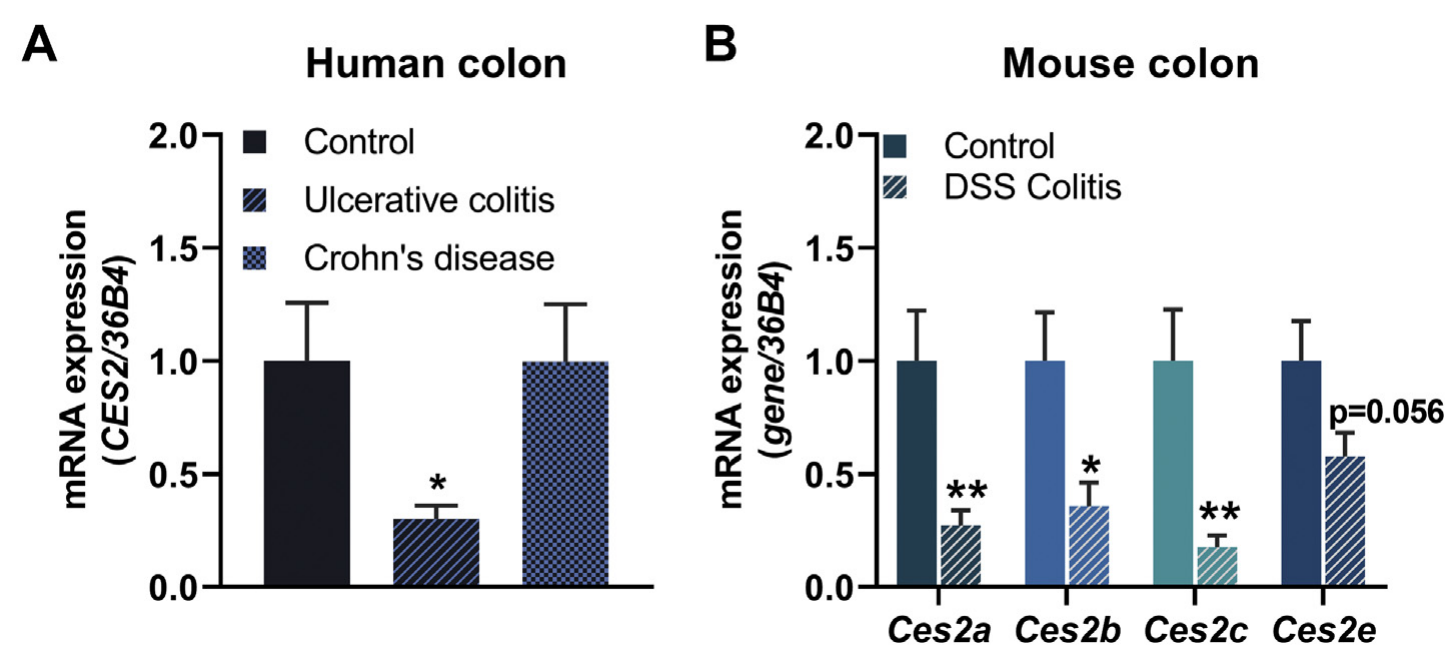
*CES2/Ces2* mRNA levels in colon biopsies derived from patients presented with inflammatory colon diseases. A: Relative mRNA expression levels of *CES2* in patients with ulcerative colitis and Crohn’s disease determined by quantitative polymerase chain reaction (qPCR) with *36B4* as the reference gene and control set to 1 as arbitrary unit (n = 7/group). B: Relative mRNA expression of *Ces2a*, *Ces2b*, *Ces2c*, and *Ces2e* in the colon of mice treated with dextran sulfate sodium (DSS) to induce colitis determined by qPCR with *36B4* as the reference gene and control set to 1 (M, *ad libitum* fed, 16 weeks of age, n = 6–7/group). Data are presented as the means ± SEM. Statistical significance was determined by unpaired Student’s *t* test (∗*P* < 0.05; ∗∗*P* < 0.01 compared with the control). CES2, carboxylesterase 2.

Because the prevalence of obesity is associated with a higher risk for the development of inflammatory bowel diseases (18, 19), we further investigated *CES2* mRNA expression levels in colon biopsies from patients suffering from ulcerative colitis and Crohn’s disease. Remarkably, *CES2* expression was significantly reduced in patients with ulcerative colitis but not in patients with Crohn’s disease (Fig. 8A). Of note, the expression of *CES1* and *CES3* was barely detectable in the human colon samples independent of the clinical status (data not shown), indicating that CES2 is the main carboxylesterase in the human colon. To evaluate whether murine *Ces2* gene members phenocopy the expression pattern of CES2 in the presence of colitis, we investigated colon samples from mice treated with DSS to induce colitis. Similar to patients suffering from colitis, mRNA expression of *Ces2a*, *Ces2b*, *Ces2c*, and *Ces2e* was either significantly downregulated or tended to be reduced (Fig. 8B). Thus, colitis is associated with reduced *CES2/Ces2* gene expression in the human and murine colons, respectively.

## Discussion

The lipase machinery involved in lipolysis from intracellular lipid droplets is comprehensively established (20). More recently, Ces1 and Ces2 family members have been elucidated as ER-associated metabolic lipases involved in the production of TG-rich lipoproteins and the regulation of lipogenesis (7). In humans, reduced *CES2* expression has been observed in liver biopsies of patients with NASH (10) and obese individuals (6). Likewise, reduced hepatic *Ces2c* expression was shown in mice kept on HFD (6, 10). These studies demonstrate TG and DG hydrolytic activities for CES2/Ces2c leading to the assumption that Ces2c is the functional ortholog of human CES2. However, biochemical characterization of murine Ces2 family members and human CES2 is incomplete. Assignment of lipid hydrolytic activities from purified Ces2/CES2 proteins and investigation of the sequential hydrolysis from TG to finally glycerol and FAs is essential for understanding the functional role of Ces2/CES2 proteins in lipid metabolism and lipid signaling.

Here we identified Ces2a, Ces2b, and Ces2e as novel TG hydrolyzing enzymes in addition to the established roles of Ces2c and CES2 as TG lipases (6, 9). Considering that TG hydrolysis delivers DG as lipid intermediate, which can be further hydrolyzed, we cannot assay specific TG hydrolytic activities with respect to FAs solely generated from TG. That said, measurement of DG and MG hydrolytic activities revealed that Ces2c, Ces2e, and, to a lesser extent, CES2 are highly active DGHs, whereas Ces2b, Ces2c, CES2, and, to a lesser extent, Ces2e are potent MG-hydrolyzing lipases markedly surpassing measured TG hydrolytic activities. Moreover, MG hydrolytic activities of all investigated Ces2/CES2 proteins even surpass measured DGH activities.

Sequence analyses of the investigated Ces2/CES2 proteins revealed a C-terminal ER retention sequence (HXEL) hindering protein secretion out of the ER lumen, which suggests a role of these lipases in ER lipid catabolism (7). The ER acts as a hub for phospholipid and TG synthesis and the generated lipids can be utilized for the formation of cytosolic lipid droplets or the incorporation into TG-rich lipoproteins in the intestine and liver. The final step in TG formation at the ER is catalyzed by the Acyl-CoA:Diacylglycerol Acyltransferase (DGAT) enzymes DGAT1 and DGAT2 (21). Deletion or knockdown of either DGAT1 or DGAT2 provokes DG accumulation at the ER, which has been tightly linked to lipotoxicity, ER stress, and the development of insulin resistance (22, 23, 24). Although yet speculative, the discovery of Ces2/CES2 proteins as highly efficient DGH and MGH suggests a role of these enzymes in the elimination of a surplus of DG and MG at the ER, thereby likely counteracting lipotoxicity and ER stress. In line with this assumption, Ruby *et al.* (6) showed substantial DG accumulation in the liver of obese individuals together with reduced CES2 expression, which might be the result of impaired CES2-mediated DG hydrolysis. Interestingly, a recent study demonstrated that pharmacological inhibition of DGAT2 and consequently DG accumulation provokes hepatic insulin resistance in mice (24). Notably, injection of a recombinant adenovirus encoding human CES2 improved insulin sensitivity in HFD-fed mice and counteracted hepatic DG accumulation, further implicating a role for CES2 in DG catabolism and lipid signaling. In accordance with this study, we also observed reduced *Ces2a* and *Ces2c* expression in the liver of HFD-challenged mice, which may promote DG accumulation typically seen in obesity. In contrast, *Ces2b* expression was strongly increased in the intestine upon HFD, whereas *Ces2a* and *Ces2c* expression was significantly reduced in colon tissue.

Low *CES2* expression linked to hepatic inflammation in humans prompted us to investigate *CES2* expression levels in colon biopsies of patients affected by inflammatory bowel disease. Intriguingly, we observed a significant decline in *CES2* mRNA expression in colon biopsies of humans affected with ulcerative colitis, whereas levels were unchanged in patients with Crohn's disease. In comparison with *CES2*, mRNA expression of *CES1* and *CES3* was extremely low in the colon, implicating that CES2 plays an important role in colon lipid catabolism and/or lipid signaling. The marked reduction in *Ces2a*, *Ces2b*, and *Ces2c* expression in the DSS-induced colitis mouse model further corroborates the assumption that CES2 is involved in the development and/or progression of inflammatory bowel disease. In recent years, the endocannabinoid system has become a promising pharmacological target for the treatment of inflammatory bowel disease, and the activation of endocannabinoid receptors has been shown to ameliorate inflammation in mouse models for ulcerative colitis (25). Interestingly, a rise of the endogenous endocannabinoid 2-AG due to pharmacological inhibition of monoacylglycerol lipase (26) also reduced gut inflammation in the colitis mouse model (27). Although speculative, CES2 could dually impact 2-AG levels either via DG hydrolysis to increase 2-AG levels or via MG hydrolysis, which is an interesting question for future studies. Yet, Ces2/CES2 proteins could also impact hepatic and intestinal lipid metabolism via the supply of FAs and lipid intermediates such as DGs and MGs for re-esterification and the production of TG-rich lipoproteins as has been shown for Ces2c (9) and other carboxylesterases including mouse Ces1d and human CES1 (28, 29).

Together, we show that mouse and human Ces2/CES2 proteins are highly active DGH and MGH that have the potential to impact lipid homeostasis and signaling in the liver and gut. Asking for the murine ortholog of human CES2, we propose that there is not a single mouse Ces2 ortholog with regard to lipid hydrolytic activities but rather orthologous functions of specific Ces2 members depending on the tissue-specific expression. This hypothesis is supported by (1) high sequence identities among all murine Ces2 proteins compared with CES2 and (2) comparable secondary structure content. Finally, this study suggests that CES2 plays a major role in DG and MG hydrolysis, which could impact not only lipid homeostasis and signaling but also therapeutic interventions with respect to the established role of CES2 in drug metabolism (16).

## Data availability

The data that support the findings of this study are listed in the article and are available from the corresponding authors upon reasonable request.

## Supplemental data

This article contains supplemental data.

## Conflict of interest

The authors declare no conflicts of interest with the contents of the articles.
