# Supplementary material for: Carboxylesterase 2 proteins are efficient diglyceride and monoglyceride lipases possibly implicated in metabolic disease
Source: J Lipid Res. 2021 Apr 17;62:100075. doi: 10.1016/j.jlr.2021.100075 (PMC8131317; doi:10.1016/j.jlr.2021.100075)
Supplement: Supplemental Figure S1 and Tables S1–S3 [file mmc1.docx]

**Supplemental Information**

**Carboxylesterase 2 proteins are efficient diglyceride and monoglyceride lipases possibly implicated in metabolic disease**

**Running title:** CES2/Ces2 proteins are potent DG and MG hydrolases

Gabriel Chalhoub^1^, Stephanie Kolleritsch^1^, Lisa K. Maresch^1^, Ulrike Taschler^1^, Laura Pajed^1^, Anna Tilp^1^, Helgit Natmessnig^1^, Philipp Rosina^1^, Benedikt Kien^1^, Franz P.W. Radner^1^, Rudolf Schicho^2^, Monika Oberer^1^, Gabriele Schoiswohl^1^*^#^, Guenter Haemmerle^1^*

**Emails**

Chalhoub, Gabriel: gabriel.chalhoub@uni-graz.at; Kolleritsch, Stephanie: stephanie.kolleritsch@uni-graz.at; Lisa K. Maresch: lisak.maresch@gmail.com; Taschler, Ulrike: ulrike.taschler@uni-graz.at; Anna Tilp: anna.tilp@edu.uni-graz.at; Pajed, Laura: laura.pajed@uni-graz.at; Natmessnig, Helgit: helgit.natmessnig@uni-graz.at; Rosina, Philipp: philipp.rosina@uni-graz.at; Kien, Benedikt: benedikt.kien@uni-graz.at; Radner, Franz: franz.radner@uni-graz.at; Schicho, Rudolf: rudolf.schicho@medunigraz.at; Oberer, Monika: m.oberer@uni-graz.at; Schoiswohl, Gabriele: gabriele.schoiswohl@uni-graz.at; Haemmerle, Guenter: guenter.haemmerle@uni-graz.at.

**Author Affiliations**

^1^Institute of Molecular Biosciences, University of Graz, 8010 Graz, Austria

^2^Division of Pharmacology, Otto Loewi Research Center, Medical University of Graz, 8010 Graz, Austria

*Correspondence: gabriele.schoiswohl@uni-graz.at (G.S.), guenter.haemmerle@uni-graz.at (G.H.)

^#^new affiliation: Department of Pharmacology and Toxicology, University of Graz, 8010 Graz, Austria

**Supplemental Tables**

**Supplemental Table 1. Primers for murine and human Ces2/CES2 constructs**

| **Primer** | **Primer Sequence** | **Restriction site** |
| --- | --- | --- |
| *Ces2a*  *Ces2a* | fw: 5'-TTA CGG GAA TTC ACC ATG CCA TTG GCT AGA CTT CC -3'  rv: 5'-CCG GTT TCT AGA CAG CTC TGC ATG CTT GTC C -3' | *EcoRI*  *XbaI* |
| *Ces2b*  *Ces2b* | fw: 5'-TTA CGG GAA TTC ACC ATG CCA CGG AGC CAA ATG -3'  rv: 5'-CCG GTT TCT AGA AAG CTC CGT GTG CTT GTC CT -3' | *EcoRI*  *XbaI* |
| *Ces2c*  *Ces2c* | fw: 5'-TTA CGG GAA TTC ACC ATG ACA CGG AAC CAA CTA CAT AAC-3'  rv: 5'-CCG GTT TCT AGA AAG CTC CCT GTG CTT GTC CT-3' | *EcoRI*  *XbaI* |
| *Ces2e*  *Ces2e* | fw: 5'-TTA CGG GAA TTC ACC ATG CCA CTA TAC AAA CTT CTT GG-3'  rv: 5'-CCG GTT TCT AGA CAA CTC TTT GTG CCT CTC CT-3' | *EcoRI*  *XbaI* |
| *Ces2f*  *Ces2f* | fw: 5'-TTA CGG GAA TTC ACC ATG CCA GTG CAC AGA CTT CCT-3'  rv: 5'-CCG GTT TCT AGA TAC AGC CTT GAT TTT ATC GTG ATT T-3' | *EcoRI*  *XbaI* |
| *Ces2g*  *Ces2g* | fw: 5’-TTG GCC GGT ACC ACC ATG CCA CGG AAC CAA ATG C-3’  rv: 5’-GGC CTT TCT AGA TAG CTC TTT GTG CAC GTT TTG AG-3’ | *KpnI*  *XbaI* |
| *Ces2h* | cDNA was synthesized by BioCat, Heidelberg, Germany | *HindIII*  *EcoRI* |
| *His-Ces2a/c*  *His-Ces2a/c* | fw: 5’-CAC CAC CAC TAG GTT GGG TGG CAT CCC-3’  rv: 5’-ATG ATG ATG AGC CCG GGA TCC TCT AGA-3’ | **-** |
| *His-Ces2e*  *His-Ces2e* | fw: 5'-ACT GAA TTC ACC ATG CCA CTA TAC-3'  rv: 5'-ATT CTA GAT TAA TGA TGA TGA TGA TGA TGC AAC TCT TTG TGC CTC TC-3' | *EcoRI*  *XbaI* |
| *His-CES2*  *His-CES2* | fw: 5'-ATC GGT ACC ACC ATG ACT GC-3'  rv: 5'-GTT CTA GAT CAA TGA TGA TGA TGA TGA TGC AGC TCT GTG TGT CTC TCT TC-3' | *KpnI*  *XbaI* |

**Supplemental Table 2. RT-PCR primers**

| **Gene** | **Primer Sequence** |
| --- | --- |
| ***Ces2a*** | fw: CTCACAGCCGGCCATGT  rv: AGATTCATTTCCTTCGCATCCT |
| ***Ces2b*** | fw: TCTGAGATGGTCTCCACTACG  rv: GCAGGGATCATCTGGACAAGC |
| ***Ces2c*** | fw: GCTGAATGCTGGGTTCTTCG  rv: GCTGCCTTGGATCTGTCCTGT |
| ***Ces2e*** | fw: CTTGTCTTTGGCTACCAGTTCG  rv: TTGCTCCTCTTCCTCAGTGTAAGG |
| ***Ces2f*** | fw: TTCAGCGTTCCCATGCTCCT  rv: CTTGAGTAAACTGGACCTATGCTG |
| ***Ces2g*** | fw: TCTCTGAGGTGGTTTACCAAACG  rv: CCTCTCAGACAGCGCACCAG |
| ***Ces2h*** | fw: AACTGTCTACGAGGCAAAAGCG  rv: GAGGATGTCTGGGCAGGAAGAT |
| ***CES1*** | fw: ACC CCT GAG GTT TAC TCC ACC  rv: TGC ACA TAG GAG GGT ACG AGG |
| ***CES2*** | fw: GTC TTC GCT TGT TGT GTC C  rv: AAC TTG GTC ACA GGC AGA CA |
| ***CES3*** | fw: CAA CAC CCG TCT TGA CCA GTC  rv: CTG GAA CGC CTG GCA TTT G |
| ***36B4*** | fw: GCT TCA TTG TGG GAG CAG ACA  rv: CAT GGT GTT CTT GCC CAT CAG |

**Supplemental Table 3. Conditions for hydrolase activity assays.**

| **Assay** | **Protein** | **Ces2a**  **µg** | **Ces2b**  **µg** | **Ces2c**  **µg** | **Ces2e**  **µg** | **CES2**  **µg** | **Substrate** | **Conc.**  **mM** | **Time**  **min** |
| --- | --- | --- | --- | --- | --- | --- | --- | --- | --- |
| MGH | purified | 0.2 | 0.1 | 0.1 | 0.2 | 0.1 | 1,3 *rac*-MO | 2 | 30 |
| DGH | purified | 2 | 2 | 0.2 | 0.5 | 0.5 | 1,2 *sn*-DO | 2 | 30 |
| DGH TLC | purified | 2 | 2 | 0.1 | 0.5 | 2 | 1,3/1,2 DO | 0.3 | 0-60 |
| TGH | purified | 0.75 | 0.75 | 0.5 | 0.5 | 0.75 | 1µCi ^3^H TO | 0.3 | 30 |
| TGH | For TGH assays performed with cell lysates, 25 µg protein was used for all investigated Ces2/CES2 proteins | | | | | | 1µCi ^3^H TO | 1.67 | 60 |

**Supplemental Figure and figure legend**


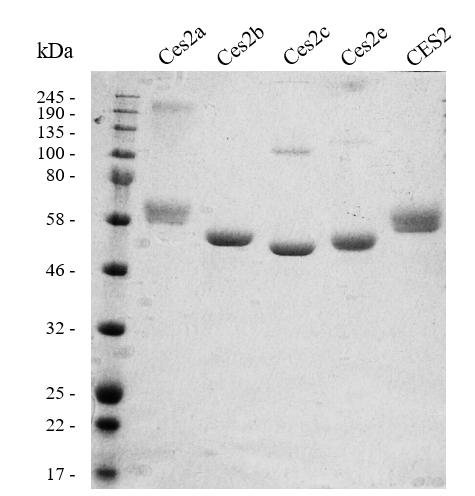


**Supplemental Figure 1. Purification of murine and human Ces2/CES2 proteins.** C-terminally His-tagged Ces2a, Ces2b, Ces2c, Ces2e, and CES2 proteins were overexpressed in Expi293F cells followed by purification with affinity chromatography. After purification, 2 µg of each protein was loaded on SDS gel and visualized with Coomassie blue stain.
